# Obesity interacts with hyperuricemia on the severity of non-alcoholic fatty liver disease

**DOI:** 10.1186/s12876-021-01615-w

**Published:** 2021-01-28

**Authors:** Mimi Zhou, Nan Yang, Xin Xing, Danyan Chang, Juan Li, Jiang Deng, Yi Chen, Chunhua Hu, Rou Zhang, Xiaolan Lu, Yingren Zhao, Yingli He

**Affiliations:** 1 Department of Infectious Diseases, The First Affiliated Hospital of Xi’an Jiaotong University, No. 277 Yanta Road (w), Xi’an, 710061 Shaanxi China; 2 Shaanxi Clinical Research Center of Infectious Diseases, Xi’an, Shaanxi China; 3 Department of Gastroenterology, Shanghai Pudong Hospital of Fudan University, Shanghai, China; 4 Department of Gastroenterology, The Second Affiliated Hospital of Xi’an Jiaotong University, Xi’an, Shaanxi China

**Keywords:** Uric acid, Obesity, Non-alcoholic fatty liver disease, Transient elastography

## Abstract

**Background:**

A series of evidence revealed that body mass index was an important confounding factor in the research of uric acid and ischemic heart disease/hypertension. The objective of this study was to investigate whether obesity status can modify the association between serum uric acid and the severity of liver damage in NAFLD, and the possible interactive effect of hyperuricemia and obesity.

**Methods:**

We conducted a cross-sectional study in a total of 557 ultrasound diagnosed-NAFLD. The hepatic steatosis and liver fibrosis were quantitatively evaluated by transient elastography. Hyperuricemia was defined as serum uric acid > 420 μmol/L in men, > 360 μmol/L in women and obesity was defined as body mass index ≥ 25 kg/m^2^. The adjusted OR values of hyperuricemia and obesity were analyzed by multivariate logistic regression analysis, and the additive model was used to investigate the possible interactive effect.

**Results:**

Multivariate regression analysis showed that hyperuricemia was associated with serious hepatic steatosis (1.74[1.09–2.79]) and elevated ALT (2.17[1.38–3.41]), but not with advanced fibrosis (1.61[0.91–2.85]). The association was further investigated in different BMI group. Hyperuricemia was associated with higher odds of serious hepatic steatosis (2.02[1.14–3.57]) and elevated ALT (2.27[1.37–3.76]) only in obese NAFLD, not in non-obese subjects. Similarly, patients with hyperuricemia had higher odds of advanced fibrosis in obese subjects (2.17[1.13–4.18]), not in non-obese subjects (0.60[0.14–2.70]). Furthermore, there was an additive interaction between hyperuricemia and obesity on the odds of serious hepatic steatosis (AP: 0.39[0.01–0.77]) and advanced fibrosis. (AP: 0.60[0.26–0.95]).

**Conclusions:**

Hyperuricemia and obesity had a significantly synergistic effect on the hepatic steatosis and fibrosis. Thus, management of uric acid may need to be targeted in obese NAFLD.

## Background

Non-alcoholic fatty liver disease (NAFLD) is the most rapidly growing chronic liver disease and epidemiological data indicates that the prevalence of NAFLD is currently 25% globally [1]. Nowadays, China is in a period of rapid growth of NAFLD, and national prevalence is up to 29.2% [2].It is commonly accepted that NAFLD can increase the risk of cirrhosis and hepatocellular carcinoma, NAFLD‐cirrhosis or NAFLD‐hepatocellular carcinoma (HCC) ranks the second in the indication for liver transplantation in the United State [3]. The annual direct medical costs are about $103 billion ($1613 per patient) in the USA and €35 billion (from €354 to €1,163 per patient) in the Europe [4]. NAFLD is closely related to metabolic syndrome and its related conditions, including obesity, type 2 diabetes, dyslipidemia, hypertension and hyperuricemia.

Previous studies have revealed an independent link between hyperuricemia and the severity of liver damage in NAFLD. A biopsy-based study has demonstrated that hyperuricemia is associated with the severity of steatosis, lobular inflammation and nonalcoholic fatty liver disease activity score (NAS) [5]. Similar findings were reported that hyperuricemia is associated with NAS score in children and adolescents [6, 7]. However, some previous studies have found a negative correlation between serum uric acid levels (SUA)and fibrosis stage [8, 9]. Whereas a meta-analysis has revealed that hyperuricemia is not associated with fibrosis in patients with NAFLD [10]. The association between SUA and fibrosis is controversial.

Obesity is an established risk factor for the occurrence and development of NAFLD. Recently, a series of evidence reveal that body mass index (BMI) is an important confounding factor in the research of uric acid and metabolic diseases. Based on the data from two large prospective cohort study, Palmer shows that uric acid has no causal effect on the risk of ischemic heart disease and blood pressure. This conclusion is inconsistent with previous studies mainly due to the confounding role of BMI [11]. The Tromsø Study from Europe reveals that baseline level of uric acid independently predicts occurrence of elevated blood pressure and elevated fasting glucose in the overweight, but not in normal-weight group, and further put forward that hyperuricemia might be treated differently in normal-weight and overweight individuals [12]. Similarly, a research based on Korean population demonstrates that the level of SUA is related to an increased risk of metabolic syndrome in non-obese population, but not in obese individuals [13].

Hence, we assume that it is of importance to take obesity status into account to investigate the association between hyperuricemia and the severity of NAFLD, particularly in the liver fibrosis. The objective of this study was to investigate whether obesity status can modify the association between serum uric acid and the severity of liver damage in NAFLD, and the possible interactive effect of hyperuricemia and obesity.

## Methods

### Participants

A total of 557 ultrasound diagnosed-NAFLD patients from the Second Affiliated Hospital of Xi'an Jiaotong University during March 2014 to January 2018 were included in this cross-sectional study. NAFLD was defined as hepatic steatosis detected by abdominal ultrasound, which was evaluated by two experienced sonographers independently. If there was an inconsistent diagnosis, the third sonographer was needed for evaluation. Exclusion criteria: (1) excessive alcohol consumption (> 20 g/day in man and 10 g/day in women) (2) use of steatogenic medications within the past 6 months (3) positive tests for hepatitis B surface antigen and hepatitis C antibody (4) drug-induced liver injury and autoimmune hepatitis (5) cirrhosis and other causes of liver disease (hemochromatosis, Wilson’s disease) [14]. Patients were divided into normouricemia group and hyperuricemia group according to the level of SUA, and further stratified into non-obese group and obese group based on BMI. Hyperuricemia was defined as SUA > 420 μmol/L in men, > 360 μmol/L  in women [15]. According to Asia–Pacific BMI criteria, obesity was defined as BMI ≥ 25 kg/m^2^ and non-obesity was BMI < 25 kg/m^2^ [16]. All patients received B-ultrasound and Fibro Touch examination at the same time. The study design was approved by the Ethics Committee of Xi'an Jiaotong University complying with Declaration of Helsinki and all participants have signed the written informed consent.

### Demographic and biochemical data of participants

Demographic data was derived from the electronic medical record, including age, gender, height, weight, blood pressure, history of hypertension/diabetes/underlying liver disease/alcohol consumption (specific alcohol consumption). Biochemical markers were as follows: alanine transaminase (ALT), fasting plasma glucose (FPG), serum uric acid (SUA), triglyceride (TG), high-density lipoprotein cholesterol (HDL-C), platelets (PLT).

### Assessment of the hepatic steatosis and fibrosis

Transient elastography (FibroTouch) was performed to assess hepatic steatosis (CAP value) and fibrosis (LSM value) quantitatively. It was conducted by a trained operator who had performed at least 500 operations. The available measurement needed to satisfy the following conditions: (1) At least 10 valid measurements were obtained, and the success rate > 60%. (2) IQR/M of controlled attenuation parameter (CAP) and liver stiffness measurement (LSM) < 30%.

The severity of NAFLD was evaluated ranging from serious hepatic steatosis, elevated ALT to advanced fibrosis. Hepatic steatosis was divided into two groups according to CAP value: Mild steatosis ≤ 265 dB/m; Serious steatosis (moderate or severe steatosis) > 265 dB/m. Elevated ALT was defined as ALT level > 40 IU/L [17, 18]. Considering that the diagnostic accuracy of LSM was affected by obesity, we applied an algorithm of a serial combination strategy of FIB-4 and LSM to distinguish NAFLD with intermediate to high risk of advanced fibrosis [19]. This algorithm was confirmed with an increase of diagnostic accuracy, compared to LSM alone [20]. FIB-4 index = (age × AST)/(PLT × ALT^1/2^).

### Definition of metabolic syndrome

Considering the close relationship between metabolic syndrome and NAFLD, the components of metabolic syndrome were adjusted in multivariate regression analysis. The diagnosis of metabolic syndrome needed to meet at least three of the following criteria [21]: (1)central obesity: BMI ≥ 25 kg/m^2^ in both genders; (2) hypertriglyceridemia: triglycerides ≥ 1.7 mmol/L, or in therapy; (3) low HDL-C: HDL-C < 1.03 mmol/L in men and < 1.29 mmol/L in women; (4) elevated blood pressure: blood pressure ≥ 130/85 mmHg, or diagnosed hypertension; (5) elevated fasting glucose: FPG ≥ 5.6 mmol/L or diagnosed type 2 diabetes.

### Statistical analysis

Continuous variables were shown as mean with standard deviation and compared using the t-test. Categorical variables were summarized as frequencies with percentages and compared by chi-square analysis. Multivariable logistic regression analysis was performed to determine the adjusted odds ratios (ORs) for serious hepatic steatosis, elevated ALT and advanced fibrosis. A method proposed by Rothman was used to test for additive interaction between hyperuricemia and obesity. The Excel software proposed by Andersson was used to quantify the amount of additive interaction, including the relative excess risk due to the interaction (RERI), the attributable proportion due to the interaction (AP) and the synergy index(S). Zero within the 95% confidence interval of RERI and AP indicated no additive interaction, and one within the 95% confidence interval of S indicated no additive interaction. SPSS 18.0 was used for statistical analysis, and P < 0.05 was considered to be significant.

## Results

### Characteristic of participants

Characteristics of patients were shown in Table 1 according to SUA concentration. The average age and BMI of all patients was 50.77 ± 13.66 years and 26.99 ± 3.18 kg/m^2^, and 61.8% of NAFLD was male. The prevalence of metabolic syndrome and hyperuricemia was 72% and 23.9%, respectively. With regards to the incidence of components of metabolic syndrome, the highest incidence was 73.8% in central obesity, followed by low HDL-C, elevated fasting glucose and hypertriglyceridemia with an incidence of 64.1%, 61.4% and 61.2%, and the lowest was 46.7% in elevated blood pressure. In terms of the severity of NAFLD, the proportion of serious hepatic steatosis, elevated ALT and advanced fibrosis were 57.6%, 34.6% and 16.7%. Compared with normouricemia group, patients in the hyperuricemia group were younger and had higher BMI and triglyceride levels, but there was no difference in the distribution of other metabolic component. Moreover, hyperuricemia group had more serious hepatic steatosis and elevated ALT, but not advanced fibrosis.

**Table 1:** Characteristics of patients according to the level of blood uric acid

	All NAFLD	Normo-uricemia	Hyper-uricemia	*P* value
Numbers, n	557	424	133	
Age (years)*	50.77** ± **13.66	52.09 ± 12.49	46.54 ± 16.20	< 0.001
Gender(male), n%	344 (61.8%)	265 (62.5%)	79 (59.4%)	0.521
BMI (kg/m^2^)*	26.99 ± 3.18	26.83 ± 3.04	27.51 ± 3.52	0.03
TG (mmol/L)*	2.37 ± 1.71	2.24 ± 1.60	2.76 ± 1.98	0.006
HDL-C (mmol/L)	1.06 ± 0.28	1.06 ± 0.28	1.07 ± 0.29	0.686
FPG (mmol/L)	7.04 ± 3.05	7.09 ± 3.03	6.88 ± 3.12	0.487
Obesity, n (%)	411 (73.8%)	306 (72.2%)	105 (78.9%)	0.121
Hypertriglyceridemia, n (%)*	341 (61.2%)	249 (58.7%)	92 (69.2%)	0.031
Low-HDL, n (%)	357 (64.1%)	272 (64.2%)	85 (63.9%)	0.960
Elevated fasting glucose, n (%)	342 (61.4%)	260 (61.3%)	82 (61.7%)	0.945
Elevated blood pressure, n (%)	260 (46.7%)	192 (45.3%)	68 (51.1%)	0.238
Metabolic syndrome, n (%)*	401 (72%)	296 (69.8%)	105 (78.9%)	0.041
CAP (dB/m)*	273.50 ± 28.99	269.78 ± 27.50	285.38 ± 30.50	< 0.001
ALT(IU/L)*	44.90 ± 43.31	39.20 ± 33.58	63.05 ± 62.08	< 0.001
FIB-4 index	1.35 ± 0.85	1.37 ± 0.79	1.30 ± 1.04	0.449
LSM (kPa)*	7.34 ± 3.17	7.09 ± 2.80	8.15 ± 4.05	0.005
Serious hepatic steatosis, n (%)*	321(57.6%)	225(53.1%)	96(72.2%)	< 0.001
Elevated ALT, n (%) *	193 (34.6%)	124 (29.2%)	69 (51.9%)	< 0.001
Advanced fibrosis n (%)	93 (16.7%)	67 (15.8%)	26 (19.5%)	0.312

Continuous variables were shows as mean ± standard deviation, and categorical variables were summarized with frequencies and percentages

*BMI* body mass index, *TG* triglyceride, *HDL-C* high-density lipoprotein cholesterol, *FPG* fasting plasma glucose, *CAP* controlled attenuation parameter, *ALT* alanine transaminase, *LSM* liver stiffness measurement

*P < 0.05 after t-test or chi-square analysis

### Obesity did modify the association between hyperuricemia and the severity of NAFLD

The adjusted ORs of hyperuricemia for the severity of NAFLD were shown in Table 2. Compared to patients with normouricemia, hyperuricemia individuals had increased odds of serious hepatic steatosis (1.74[1.09–2.79]) and elevated ALT (2.17[1.38–3.41]), not of advanced fibrosis (1.61[0.91–2.85]). Furthermore, we investigated the associations between hyperuricemia and the severity of NAFLD in different BMI group. Multivariate regression analysis showed hyperuricemia was associated with higher odds of serious hepatic steatosis (2.02[1.14–3.57]) and elevated ALT (2.27[1.37–3.76])) in obese NAFLD, not in non-obese subjects. Surprisingly, patients with hyperuricemia had higher odds of advanced fibrosis in obese subjects (2.17[1.13–4.18]), not in non-obese subjects (0.60[0.14–2.70]).

**Table 2:** Obesity did modify the association between hyperuricemia and the severity of NAFLD

	All NAFLD		*P* value	Non-obesity		*P* value	Obesity		*P* value
	Normo-uricemia	Hyper-uricemia		Normo-uricemia	Hyper-uricemia		Normo-uricemia	Hyper-uricemia	
Hepatic steatosis	1	1.74 (1.09–2.79)	0.020	1	1.56 (0.61–3.95)	0.351	1	2.02 (1.14–3.57)	0.016
Elevated ALT	1	2.17 (1.38–3.41)	0.001	1	2.76 (0.86–8.86)	0.087	1	2.27 (1.37–3.76)	0.002
Advanced fibrosis	1	1.61 (0.91–2.85)	0.100	1	0.60 (0.14–2.70)	0.508	1	2.17 (1.13–4.18)	0.020

Adjusted for age, gender, BMI, TG, low HDL-C, elevated fasting glucose, elevated blood pressure

### The individual and combined associations of hyperuricemia and obesity with the severity of NAFLD

Furthermore, patients were categorized into four groups according to the level of SUA and BMI: Control group (normouricemia and non-obesity). Hyperuricemia group (hyperuricemia and non-obesity), Obese group (normouricemia and obesity), Obese-hyperuricemia group (hyperuricemia and obesity). Compared with the control group, the ORs for the severity of liver damage in NAFLD were shown in Table 3. After adjusting for age, gender, TG, low HDL-C, elevated fasting glucose, elevated blood pressure, we found that patients in obese group had higher ORs for serious hepatic steatosis (3.32[2.09–5.28]) and elevated ALT (1.88[1.09–3.23]), not in hyperuricemia group (serious hepatic steatosis, 1.53[0.64–3.65]; elevated ALT, 2.13[0.77–5.93]). When hyperuricemia and obesity were present together, the ORs increased significantly to 6.32 and 4.01, respectively. Similarly, there was no increase in the OR for advanced fibrosis (0.61[0.15–2.42]) in the hyperuricemia group. Whereas the OR of the obese-hyperuricemia group for advanced fibrosis was obviously higher than that of obese group (4.36[1.94–9.76] vs 2.13[1.09–4.15]).

**Table 3:** The individual and combined associations of hyperuricemia and obesity with the severity of NAFLD

	Serious hepatic steatosis, n (%)		Elevated ALT, n (%)		Intermediate to high risk of advanced fibrosis, n (%)	
	OR (95% CI)	*P* value	OR (95% CI)	*P* value	OR (95% CI)	*P* value
Group A	1		1		1	
Group B	1.53 (0.64–3.65)	0.343	2.13 (0.77–5.93)	0.148	0.61 (0.15–2.42)	0.478
Group C	3.32 (2.09–5.28)	< 0.001	1.88 (1.09–3.23)	0.022	2.13 (1.09–4.15)	0.027
Group D	6.32 (3.36–11.89)	< 0.001	4.01 (2.11–7.63)	< 0.001	4.36 (1.94–9.76)	< 0.001

Adjusted for age, gender, TG, low HDL-C, elevated fasting glucose, elevated blood pressure. Group A: normo-uricemia and non-obesity. Group B: hyperuricemia and non-obesity. Group C: normo-uricemia and obesity. Group D: hyperuricemia and obesity

### Additive interaction effect between hyperuricemia and obesity on the severity of NAFLD

**Fig. 1:**
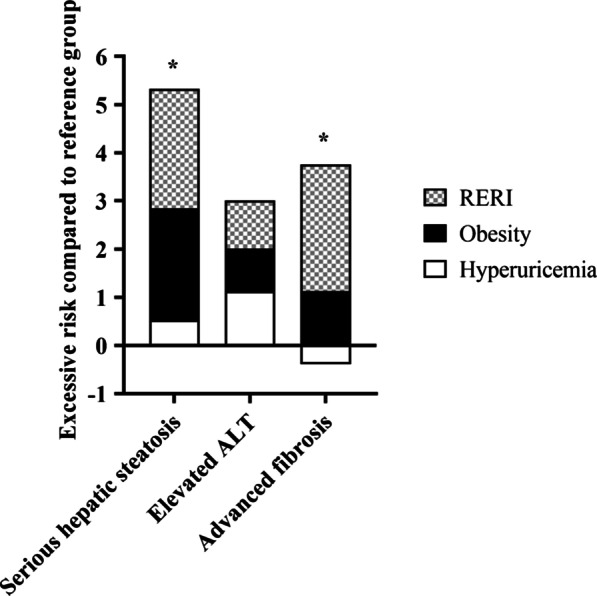
Additive interaction effect between hyperuricemia and obesity on the severity of NAFLD. RERI, the relative excessive risk due to the interaction. Reference group: normouricemia and non-obesity. *Indicated an additive interaction

We further analyzed the additive interaction effect between hyperuricemia and obesity on the severity of NAFLD, and the results were shown in Table 4 and Fig. 1. There was an additive interaction between hyperuricemia and obesity on the prevalence of serious hepatic steatosis and advanced fibrosis. (serious hepatic steatosis, AP:0.39[0.01–0.77]; advanced fibrosis, AP: 0.60[0.26–0.95]). In other words, when hyperuricemia and obesity were present together, 39% of the prevalence for serious hepatic steatosis and 60% of that for advanced fibrosis was due to the interaction effect. However, there was no evidence of an additive interaction between hyperuricemia and obesity for elevated ALT.

**Table 4:** Additive interaction effect between hyperuricemia and obesity on the severity of NAFLD

	RERI	AP	S
Serious hepatic steatosis	2.48 (− 1.01–5.96)	0.39 (0.01–0.77)*	1.87 (0.84–4.15)
Elevated ALT	1.00 (− 1.63–3.62)	0.25(− 0.35–0.85)	1.50 (0.46–4.88)
Advanced fibrosis	2.63 (− 0.16–5.41)	0.60(0.26–0.95)*	4.59 (0.57–37.20)

Adjusted for age, gender, TG, low HDL-C, elevated fasting glucose, elevated blood pressure

*Indicated an additive interaction

## Discussion

In the present study, we investigated whether obesity status can modify the association between serum uric acid and the severity of liver damage in NAFLD. There were three main findings. Firstly, hyperuricemia was associated with the severity of steatosis, liver inflammation in NAFLD, but not with liver fibrosis. Secondly, hyperuricemia was associated with higher odds of serious hepatic steatosis, elevated ALT and advanced fibrosis in obese NAFLD, whereas the significance was lost in non-obese individuals. Thirdly, there was an additive interaction between hyperuricemia and obesity on the odds of serious hepatic steatosis and advanced fibrosis.

As mentioned previously, hyperuricemia was associated with the severity of steatosis, lobular inflammation in NAFLD, but not with liver fibrosis [5, 10].These findings were consistent with ours, we found that hyperuricemia individuals had higher odds of serious hepatic steatosis and elevated ALT (serious hepatic steatosis, 1.74[1.09–2.79], elevated ALT, 2.17[1.38–3.41]), but not of advanced fibrosis (1.61[0.91–2.85]). Considering that BMI was an important confounding factor in the research of uric acid and metabolic diseases, the association between hyperuricemia and the severity of NAFLD was further explored in non-obese and obese individuals. Surprisingly, the association was significantly changed in different BMI group. In non-obese individuals, we found no evidence of association of hyperuricemia with either serious hepatic steatosis, elevated ALT or advanced fibrosis. Inversely, hyperuricemia was associated with higher odds of both serious hepatic steatosis, elevated ALT and advanced fibrosis in obese NAFLD. Our findings were line with those in other metabolic diseases. A population-based study demonstrated that hyperuricemia significantly increased the risk of hypertension only in obese individuals [22]. Likewise, asymptomatic hyperuricemia was relevant to cardiometabolic risk in obese but not in lean subjects [23]. Our results provided a possible explanation for the controversial phenomenon in the association between SUA and liver fibrosis [8–10]. This variation, to some extent, may be due to the different distribution of obesity in the study population.

When obesity and hyperuricemia were present together, there was a strikingly high odds of serious steatosis, elevated ALT and advanced fibrosis, compared with the effect of obesity alone. Whereas hyperuricemia alone was not related to the severity of liver damage. Furthermore, additive interaction analysis found that hyperuricemia and obesity had a significantly synergistic effect on the hepatic steatosis and fibrosis. Specifically, when hyperuricemia and obesity were present together, 39% of the prevalence for serious hepatic steatosis and 60% of that for advanced fibrosis was due to the interaction effect. The classical ‘two hits thesis’, commonly accepted hypothesis of NAFLD pathogenesis, may explain the interaction effect. Obesity was the ‘first hit’ which promoted lipid accumulation in liver, and increased the susceptibility of the liver to injury. Hyperuricemia, as the ‘second hits’, contributed to inflammation with hepatocyte injury and further lead to NASH and hepatic fibrosis [24]. Data in the literature suggested that uric acid can function as a powerful antioxidant to resist the oxidative stress associated with aging and cancer [25]. On the other hand, emerging evidences show that uric acid has a pro-inflammatory role in some metabolic disorders, such as obesity, hypertension, metabolic syndrome (Mets), non-alcoholic fatty liver disease (NAFLD) and cardiovascular disease [26]. Several lines of evidence reported that the hydrophobic condition of lipid altered the antioxidant property of uric acid and the oxidized lipids could turn uric acid into an oxidant [27]. Also, in the process of 3T3-L1 cells differentiated into adipocytes, supplementation of uric acid could increase ROS production by activating NADPH oxidase [28]. Thus, we speculated that uric acid played dual role of anti-oxidant and pro-oxidant in the development of NAFLD, and this dual role may influence by the obesity status. In other words, in non-obese status, uric acid may act as an anti-oxidant and it turned into pro-oxidant in the obesity status. And hyperuricemia was synergistic with obesity to exacerbate the progression of NAFLD. Therefore, we tentatively put forward that hyperuricemia should be controlled in obese NAFLD.

Here, we highlighted that uric acid may act as a pro-oxidant in the obese state, and hyperuricemia was synergistic with obesity to exacerbate the progression of NAFLD. Up to date, treatment of asymptomatic hyperuricemia was ambiguous, and optimal management of hyperuricemia was a matter of urgency in cardiovascular, metabolic, and renal comorbidities. Our study provided an idea for the targeted management of hyperuricemia in obese NAFLD. Several limitations should be noted in this study. Firstly, this was a cross-sectional study that could not determine the causal relationship between obesity, hyperuricemia and liver damage in NAFLD, only provide evidence of relevance. Thus, more large-scale prospective studies are demanded to verify results in this study. Secondly, liver biopsy was not available in this study, due to the low acceptability of liver biopsy in NAFLD. There were two main imaging techniques to non-invasively assess the degree of liver steatosis and fibrosis: ultrasound-based and magnetic resonance-based elastography techniques. MRE showed the highest diagnostic accuracy in the detection of advanced fibrosis in NAFLD. And diffusion-weighted MRI (DW-MRI) was the only functional imaging technology that can measure the motion of water in the extracellular space in living tissues, and the measured apparent diffusion coefficient (ADC) value can relatively accurately reflect the degree of liver fibrosis [29–31]. Transient elastography (TE) was second only to magnetic resonance elastography (MRE) and showed good consistency with the pathological findings by biopsy in NAFLD in assessing liver fibrosis [32]. TE had the advantages of being cheap, radiation-free and accessible, so it was applied extensively in clinical practice. Thirdly, the sample size of this study was limited, the association of hyperuricemia with the severity of NAFLD was not evaluated in different genders. But in the multivariate analysis, we adjusted gender as a confounding factor. Thus, more large-scale prospective studies are warrant.

## Conclusions

Our results revealed that hyperuricemia was associated with the severity of liver damage in obese NAFLD, not in non-obese individuals. And hyperuricemia and obesity had a significantly synergistic effect on the hepatic steatosis and fibrosis. Thus, management of uric acid may need to be targeted in obese NAFLD.

## Data Availability

The datasets used and/or analyzed during the current study are available from the corresponding author on reasonable request.

## Abbreviations

NAFLD: Non-alcoholic fatty liver disease
OR: Odds ratio
ALT: Alanine transaminase
HCC: Hepatocellular carcinoma
NAS: Nonalcoholic fatty liver disease activity score
SUA: Serum uric acid
BMI: Body mass index
CAP: Controlled attenuation parameter
LSM: Liver stiffness measurement
HDL-C: High-density lipoprotein cholesterol
FPG: Fasting plasma glucose
PLT: Platelet
RERI: Relative excess risk due to the interaction
AP: Attributable proportion
S: Synergy index
TG: Triglyceride
NASH: Nonalcoholic steatohepatitis
Mets: Metabolic syndrome
TE: Transient elastography
MRE: Magnetic resonance elastography
DW-MRI: Diffusion-weighted MRI
ADC: Apparent diffusion coefficient
